# Supplementary material for: Engineering the temporal dynamics of all-optical switching with fast and slow materials
Source: Nat Commun. 2023 Sep 21;14:5877. doi: 10.1038/s41467-023-41377-5 (PMC10514334; doi:10.1038/s41467-023-41377-5)
Supplement: Supplementary file 1 — Supplementary information [file 41467_2023_41377_MOESM1_ESM.pdf]

Supplementary Information for "Engineering the Temporal Dynamics of All-Optical Switching with *Fast* and *Slow* Materials"

S1. Growth of titanium nitride on silicon

The TiN is grown by DC reactive magnetron sputtering using a titanium target. A 99.5% pure Ti target of 2-inch diameter is used. The distance from the target to the source is kept at 20 cm to ensure uniformity. The chamber is pumped down to 10^{-8} T to prevent oxygen contamination and is backfilled with Ar at a pressure of 5 mTorr. The Ti is sputtered for 2 minutes to clean the top surface with a power of 200W. Then, an Ar:N_2 mixture of 1:18 ratios is used for the sputtering process. The substrate is heated to a temperature of 800°C and rotated at 5 rpm.

S2. Growth of aluminum-doped zinc oxide

We grow the AZO on optically thick TiN layers grown on silicon using the method mentioned above. The substrates are heated at 45°C. The chamber is pumped down to a vacuum of 10^{-6} Torr. ZnO is deposited on the TiN films by pulsed laser deposition (PLD) with a KrF excimer laser at a pump fluence of 1.5 J/cm^2. The chamber is pumped down to 6×10^{-6} T and then backfilled to 3 mTorr with oxygen. The growth rate is 6 nm/min. We also grow AZO on silicon substrates and fused silica using the same growth parameters.

S3. Steady-state optical properties of titanium nitride and aluminum doped zinc oxide

The TiN films are measured with the variable angle spectroscopic ellipsometry (VASE) at angles of 50 and 70°. We used a Drude Lorentz model to describe the optical properties of the TiN, with one Drude oscillator and two Lorentz oscillators.

$$\varepsilon = \varepsilon_1 + i\varepsilon_2 = \varepsilon_\infty - \frac{A_0}{(\hbar\omega)^2 + iB_0\hbar\omega} + \sum_k^n \frac{A_k}{E_k^2 - (\hbar\omega)^2 - iB_k\hbar\omega} \quad \text{(Eq. S1)}$$

where ε_1 and ε_2 are the real and imaginary parts of the dielectric permittivity, ħ is the reduced Plank constant, ω is the probe angular frequency, and ε_∞ is an additional offset. The negative (Drude) part shows the contribution of the free carriers to the permittivity: A_0 is the square of the plasma frequency (in eV) and B_0 is the damping factor of the Drude oscillator. The effect of bound electrons and interband transitions is modeled by a summation of the Lorentz oscillators, with A_k, B_k, and E_k being the amplitude, broadening, and center energy of individual Lorentz oscillators.

Table S1. Drude-Lorentz parameters for the as-deposited TiN and AZO films

Thickness	A_0	B_0	A_1	B_1	E_1	A_2	B_2	E_2	ε_∞
TiN	43.3	0.196	310.6	76.2	4.61	31.6	0.993	4.15	3.64
AZO on TiN	2.91	0.139	17.8	0.147	4.55	-	-	-	2.54
AZO on Si	3.03	0.086	4.52	0.122	4.27				3.08
AZO on FS	2.80	0.079	309	50.6	26.6	-	-	-	2.88

S4. Temporal dynamics of TiN

Fig. S1 shows the temporal dynamics of TiN when pumped with 325 nm light at a pump fluence of 1.5 mJ/cm^2. Prior work by George et al. and Diroll et al[1,2] show TiN to have an ultrafast electron-phonon relaxation rate that is see when probed near the ENZ point. Our experiment shows similar results, as seen from the sharp initial decay of the probe at 505 nm followed by the slower decay (black curve). Far from the ENZ, the ultrafast component is no longer seen, and the slower components span a nanosecond timescale (green graph). For this study, we focused on the slower dynamics of the TiN films. The overall dynamics is fitted with a two time-constant model, comprising of two exponential components, with time constants

of the faster component of ≈20 ps for TiN followed by a slower time constant of ≈250 ps, both attributed to lattice cooling via diffusion and the radiation of heat from the measured sample spot, including through available interfaces.

$$\left(\frac{\Delta R}{R}\right)(t) = Ae^{\frac{-t}{\tau_1}} + Be^{\frac{-t}{\tau_2}} + C \qquad \text{(Eq. S2)}$$

where $A,\ B,\ C, \tau_{1,2}$ are the relaxation fitting constants summarized in Table S2.

Table S2. Fitting parameters of TiN transient reflectance modulation

Wavelength (nm)	A	B	C	τ_1 (ps)	τ_2 (ps)
505	0.33	0.26	0.096	17.3	287
750	0.29	0.28	0.24	24.3	242

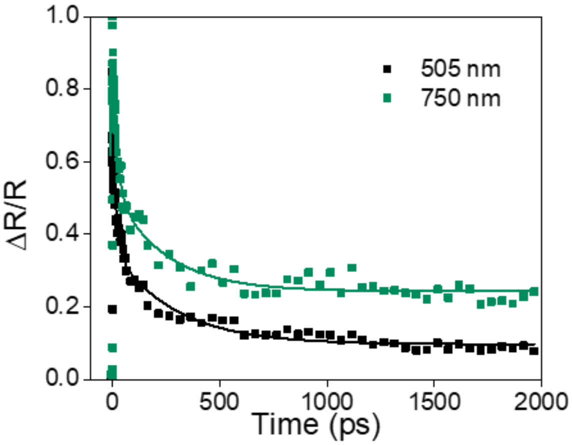

Figure S1. Normalized transient reflectance of TiN at different wavelengths

S5. Temporal dynamics of AZO on Si with different powers, and that of AZO on fused silica

We perform pump-probe spectroscopy on the AZO films at various pump fluences. Fig. S2a shows the normalized reflectance spectra of AZO at various pump fluences. The overall decay time is invariant with

increased pump fluence. Prior studies attribute this ultrafast response time to defect-assisted Shockley-Read-Hall recombination, a common recombination pathway in heavily doped TCOs [3–5].

The carrier relaxation time of doped oxides may also be dependent on the growth substrate [6]. To study how much the recombination time depends on the substrate, we grew AZO fused silica. Pump probe spectroscopy of the AZO grown on fused silica shows it to have a recombination time under 10 ps.

$$\left(\frac{\Delta R}{R}\right)(t) = D\mathrm{e}^{\frac{-t}{\tau}} + E \qquad \text{(Eq. S3)}$$

where $D,\ E,\ \tau$ are the relaxation fitting constants summarized in Table S3.

Table S3 Fitting parameters of AZO transient reflectance modulation at a wavelength of 1.2 μm

Pump Fluence (mJ/cm^2)	D	E	τ (ps)
0.18	1.76	0.056	0.715
0.49	1.69	0.046	0.873
1.53	1.98	0.045	0.723
2.48	1.65	0.088	0.834

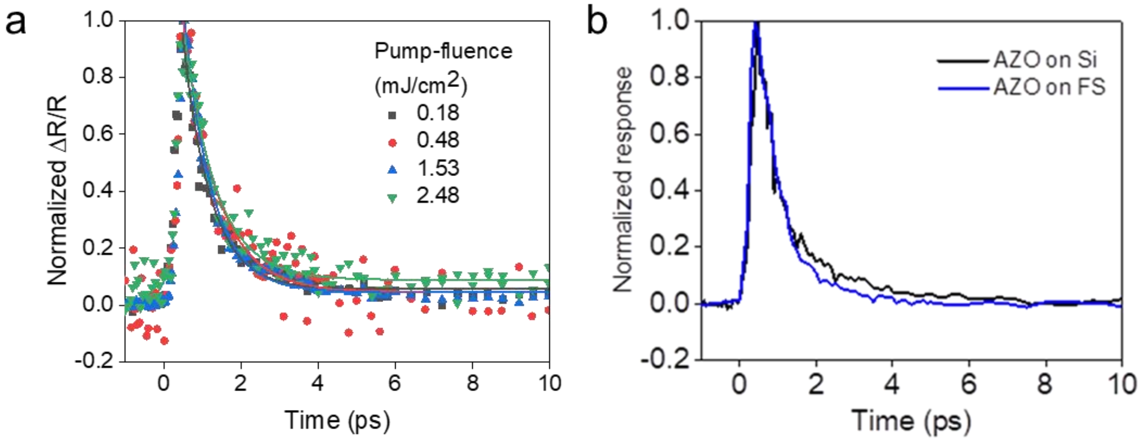

Figure S2. (a) Transient reflectance spectra of AZO on Si at different pump-fluences at 1200 nm (b) Transient reflectance spectrum of AZO on Si overlapped on that of AZO grown on fused silica, showing similar response times.

S6. Metasurface response as a weighted sum of the material responses

Having extracted the temporal responses of the individual films, the response of the metasurface at a specific wavelength can be modeled as a weighted sum of the temporal response of the TiN and the AZO films.

$$\left(\frac{\Delta R}{R}\right)(t) = \alpha\left(A\mathrm{e}^{\frac{-t}{\tau_1}} + B\mathrm{e}^{\frac{-t}{\tau_2}}\right) + \beta D\mathrm{e}^{\frac{-t}{\tau}} + \Gamma \qquad \text{(Eq. S4)}$$

α is a weight factor of TiN (with time constants, $\tau_1 = 17.3\ ps$, $\tau_2 = 287\ ps$ and constants $A = 0.33, B = 0.26$, from Table S.2 at 505 nm) and β is a weight factor of the AZO (with time constant $\tau = 0.72\ ps$ and constant $D = 1.98$ from Table S.3 at a fluence of 0.18mJ/cm^2). Γ comprises much slower lattice cooling effects, together with detector noise arising from probe fluctuations. Figure S3 shows the fitted graphs of the experimental data at each wavelength. For these fits, we used the time constants of TiN at 505 nm and of AZO at 1200 nm. Incorporating the wavelength dependent variations of the time constants can be expected to give better fits. Fig. S3b shows the relative weight of the contribution of TiN to the overall response $\alpha/(\alpha+\beta)$ (red curve)**,** overlapped with the simulated absorbance of the TiN (Abs_{TiN}) relative to the total absorbance ($Abs_{TiN+AZO}$) at each wavelength. The trends show a strong correlation. The absorbance of probe in a medium is strongly affected by the light-matter interaction between the probe and the medium. This shows that the time constants of the recombination are indeed influenced strongly by the light-matter interaction of the probe with the individual layer.

Table S4 Fitting parameters for the metasurface dynamic response

Wavelength (nm)	508	600	750	900	1180	1300
α	0.837	0.583	0.380	0.347	0.008	-0.018
β	0.476	0.702	0.965	1.147	1.338	1.045
Γ	0.08	0.135	0.001	0.050	0.023	-0.015
Reduced chi-square	5.04E-4	8.27E-4	10.7E-4	12.4E-4	4.36E-4	19.3E-4

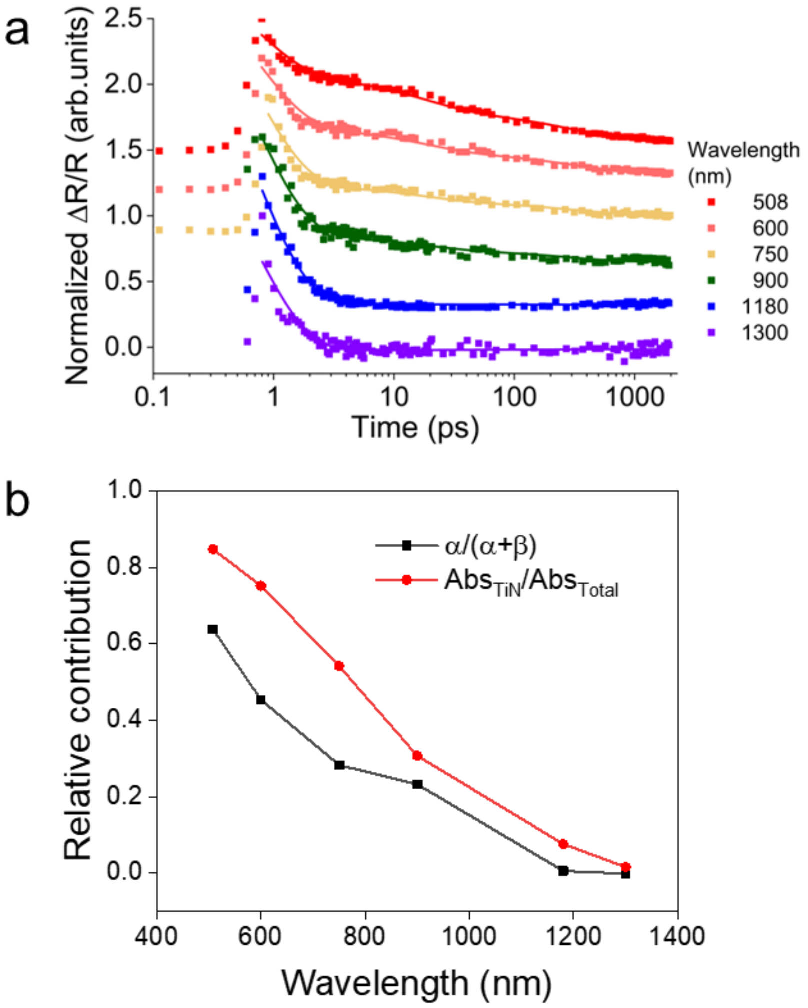

Figure S3 (a) Experimentally obtained temporal response of the metasurface (normalized) at different wavelengths (dots) overlapped with the fits (solid lines). The maximum pump-probe overlap for each experiment was alligned to center at 1 ps, and each curve is vertically translated by 0.3 from the next for clarity. (b) Relative weight of the TiN contribution to the sum of TiN and AZO contributions (red), and the relative absorbance of TiN as a fraction of the total absorbance of the metasurface (black)

S7. Controlling the device switching speed by changing the probe angle of incidence, polarization, and pump wavelength.

In our system, for a fixed wavelength, changing the **probe angle of incidence** decreases the proportion of light interacting with the fast (AZO) material and increases the interaction with the slow (TiN) material, thus slowing the effective switching rate down (Fig. S4a). In Fig. S4a, for the 30° angle of incidence, more of the electromagnetic field interacts with TiN, the dynamics thus follow the slower TiN dynamics, which take several nanoseconds to eventually fall to zero. For the 30° case, the non-zero term takes several nanoseconds to fall to zero because of the slow TiN response[1].

Similarly, we also observe a nanosecond tail in the transient response with an **s-**polarized probe at the NIR wavelength range (Fig. S4b). The sign reversal and the increase after 1 ps result from the shift and the broadening of additional Fabry-Perot resonances due to the injection of photoexcited hot-carriers, which interact with the reflectance signal. The complex interplay of resonance shift and broadening that result in the tens of picosecond to hundred picosecond components likely arises from hot carrier injection as well as thermal expansions that alter resonant conditions. In this case, s-polarization presents higher sensitivity to these changes. A more rigorous investigation of similar dynamics in ENZ films is reported in the work by Fruhling et al [7].

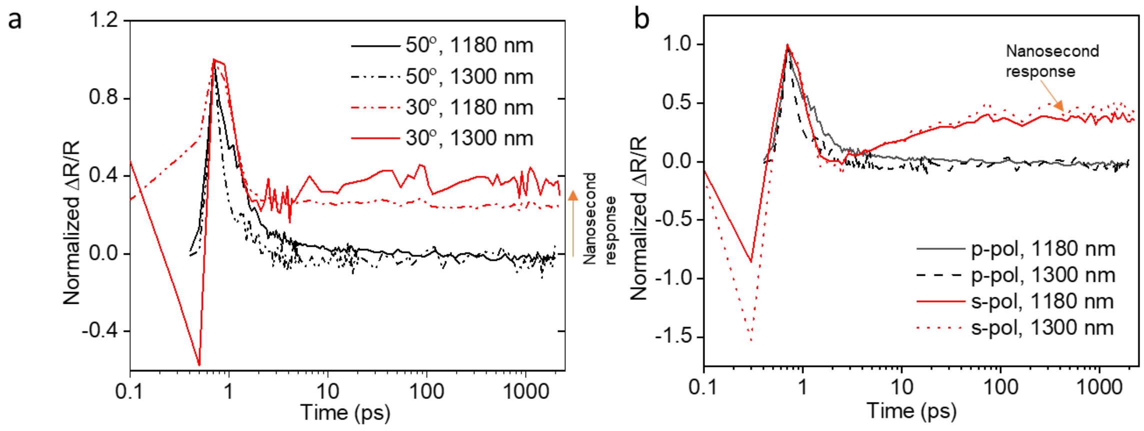

Figure S4. (a) At an angle of 30 degrees, a greater proportion of the probe interacts with the TiN, resulting in a nanosecond tail (b) A similar, slower response can also be seen by moving from a p-polarized to an s-polarized probe at the same wavelength. There is also a sign reversal of the signal change that can be attributed to complex, non-equilibrium dynamics of photoinjected carriers that result in the shift and broadening of additional resonances in the structure.

At the visible wavelengths, where most of the light will primarily interact with the TiN layer, a fast response can still be achieved by utilizing a different pump which interacts more strongly with the faster (AZO) material, thus leading to a sub-picosecond response. The following experiment shows the device response with a visible probe at 488 nm wavelength (at 50° angle of incidence) when pumped with an intraband pump of 1400 nm wavelength (p-polarized, 70° angle). At this configuration, the AZO absorbs most of the signal, resulting in a fast, femtosecond response (Fig. S5, solid black line). Note that the TiN response time at the same probe wavelength is in nanoseconds, regardless of the pump wavelength (dashed lines).

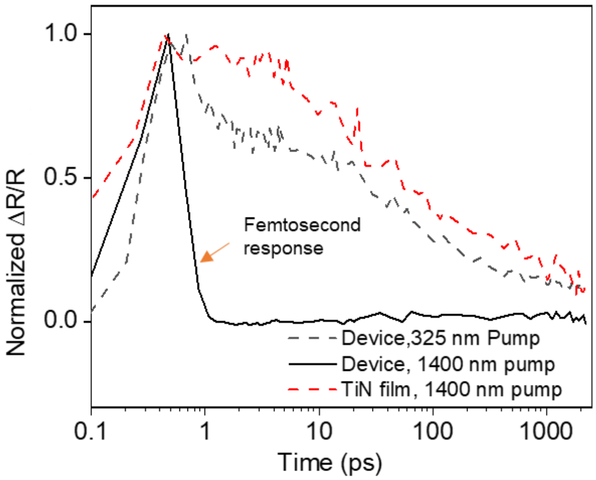

Figure S5. An intraband pump at 1400 nm and a 70° angle of incidence results in a femtosecond device response to a visible probe at 488 nm. This is because the pump is mostly absorbed in the AZO layer, resulting in a femtosecond device response, even though most of the probe interacts with the TiN layer. The dashed lines show the responses of the TiN film (red) with a 1400 nm pump, showing the slower dynamics, and the device dynamics with a 325 nm pump. “

Supplementary References

1. George, H. *et al.* Nonlinearities and carrier dynamics in refractory plasmonic TiN thin films. *Opt. Mater. Express* **9**, 3911 (2019).
2. Diroll, B. T., Saha, S., Shalaev, V. M., Boltasseva, A. & Schaller, R. D. Broadband Ultrafast Dynamics of Refractory Metals: TiN and ZrN. *Adv. Opt. Mater.* **8**, 2000652 (2020).
3. Saha, S. *et al.* Broadband, High-Speed, and Large-Amplitude Dynamic Optical Switching with Yttrium-Doped Cadmium Oxide. *Adv. Funct. Mater.* **30**, 1908377 (2020).
4. Kinsey, N. *et al.* Epsilon-near-zero Al-doped ZnO for ultrafast switching at telecom wavelengths. *Optica* **2**, 616–622 (2015).
5. Saha, S. *et al.* Extraordinarily large permittivity modulation in zinc oxide for dynamic nanophotonics. *Mater. Today* **43**, 27–36 (2021).
6. Ribut, S. H., Che Abdullah, C. A. & Mohammad Yusoff, M. Z. Investigations of structural and optical properties of zinc oxide thin films growth on various substrates. *Results Phys.* **13**, 102146 (2019).
7. Fruhling, C., Ozlu, M. G., Saha, S., Boltasseva, A. & Shalaev, V. M. Understanding all-optical switching at the epsilon-near-zero point: a tutorial review. *Appl. Phys. B Lasers Opt.* **128**, 1–12 (2022).
